# A case report of a geriatric patient with multiple-site hemorrhage due to extraosseous Ewing sarcoma

**DOI:** 10.3389/fonc.2026.1790668

**Published:** 2026-05-21

**Authors:** Shumei Song, Mingyu Zhao, Shixin Li, Shuai Zhang, Shan Zhang, Runxuan Du, Zhenguo Nie, Jianhua Zhai

**Affiliations:** 1 Department of Geriatrics Medicine, Tianjin Union Medical Center, The First Affiliated Hospital of Nankai University, Tianjin, China; 2 Department of Neurosurgery, Tianjin Medical University General Hospital, Tianjin, China

**Keywords:** extraosseous Ewing sarcoma, gastrointestinal hemorrhage, jejunal neoplasms, multidisciplinary treatment, old patient

## Abstract

Extraosseous Ewing sarcoma (EES) is a rare subtype of the undifferentiated small round cell sarcoma of bone and soft tissue. Its clinical presentation is non-specific, often manifesting initially as a local mass, pain, or systemic symptoms, such as fever and fatigue. When multisite hemorrhage serves as the initial or predominant manifestation, it is highly prone to confusion with other hemorrhagic disorders, leading to diagnostic delays. This article analyzes the diagnostic and therapeutic course of a 70-year-old patient with EES who presented with multisite hemorrhage. This report summarizes the pathogenesis, pathological characteristics, and recent advances in the treatment of EES, thereby providing a reference for timely and effective interventions.

## Introduction

Extraosseous Ewing sarcoma (EES) is a small, round cell, primary malignant soft tissue tumor that predominantly occurs in children and adolescents, and rarely occurs in elderly patients. This tumor is characterized by high invasiveness, frequent metastasis, and a poor prognosis (1). Owing to its rarity and non-specific clinical manifestations, early recognition is challenging and often leads to misdiagnosis or missed diagnosis, which may delay optimal treatment. This article reports the case of a 70-year-old male patient admitted with “intermittent melena accompanied by right lumbar back pain for over one month,” who was initially diagnosed with gastrointestinal bleeding and severe anemia. Imaging revealed a space-occupying lesion in the proximal jejunum, which was confirmed by surgical pathology as EES of the small intestine. Despite aggressive multidisciplinary interventions, including surgical resection, blood transfusion support, anti-infection therapy, and interventional treatment, the patient ultimately succumbed to late-stage tumor-related fatal hemorrhage, shock, and respiratory failure involving multiple sites (gastrointestinal, peritoneal, and respiratory). This report aims to enhance the clinical recognition and management of EES in elderly patients, particularly in atypical cases presenting with hemorrhage as the initial symptom. This report analyzes the clinical features, diagnostic challenges, and multidisciplinary collaboration process in this rare case, and is supplemented by a literature review.

## Case description

**Figure 1:**
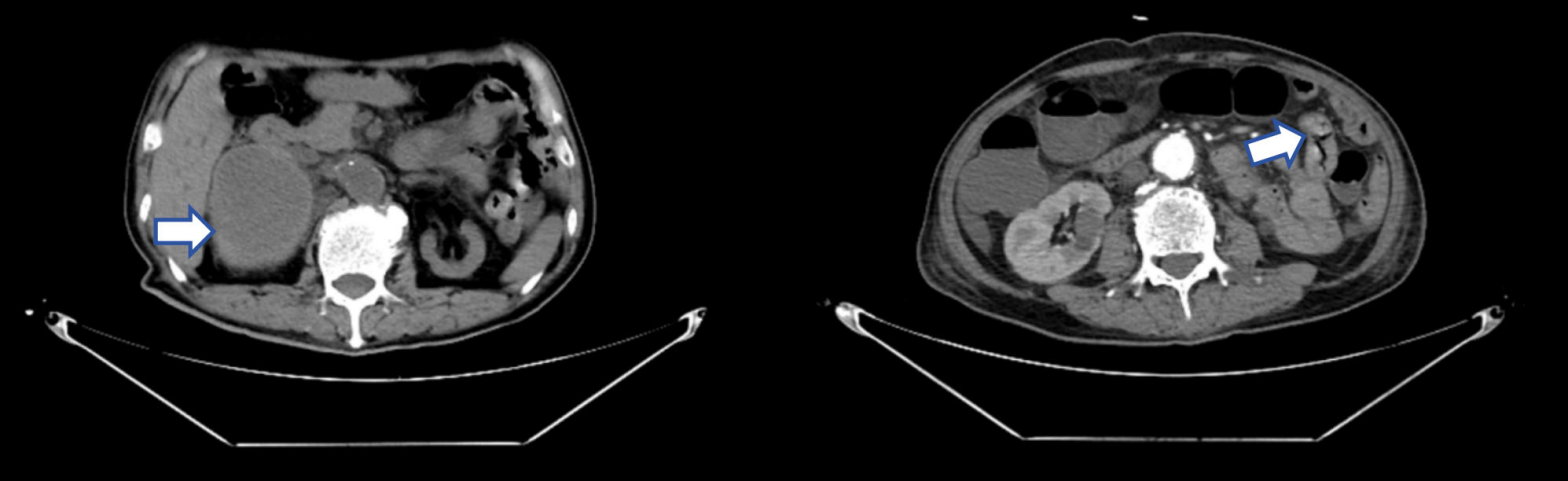
Abdominal CT image before surgery. A mass with a maximum cross-sectional size of approximately 4.0cm×3.0cm can be seen in the proximal jejunum, with mild enhancement. A mass shadow can be seen in the right adrenal area, with the maximum cross-sectional size approximately 12.3cm×8.4cm, and mild heterogeneous enhancement.

**Figure 2:**
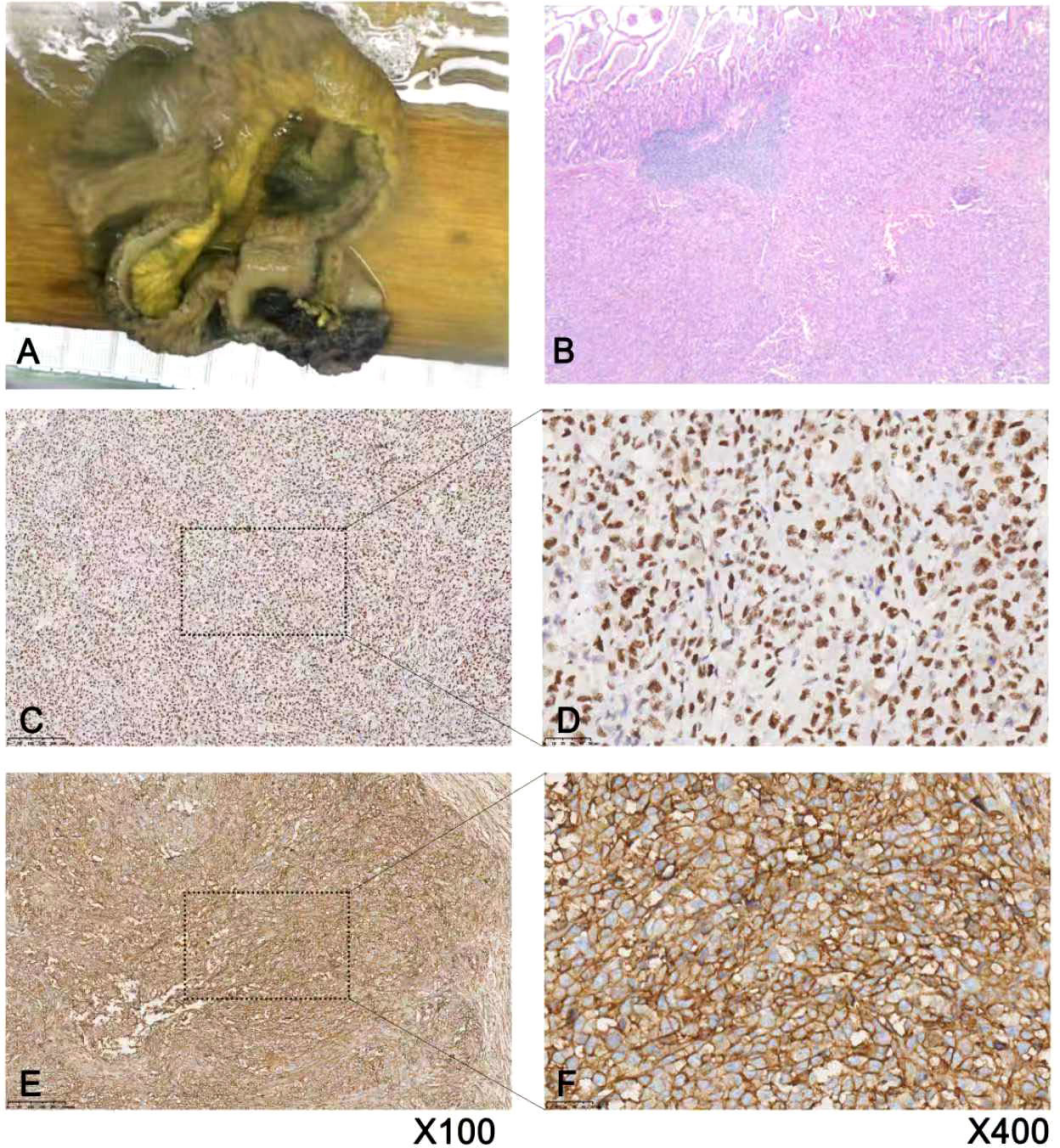
Surgical specimens and postoperative pathological microscopic manifestations of the patient. **(A)** Surgical resection specimen, jejunal tumor with hemorrhagic ulcer; **(B)** Histopathological examination revealed uniform morphology, densely arranged cells in patches, with round or oval nuclei, deeply stained nuclei, and scarce cytoplasm; **(C, D)** Nuclear positive for FLI1; **(E, F)** CD99 cell membrane diffuse strong positive.

A 70-year-old man was admitted to the hospital with a chief complaint of “intermittent melena accompanied by right lumbar back pain for over one month.” He had a history of grade 2 hypertension (high-risk). Physical examination upon admission revealed an anemic appearance, positive percussion pain in the right renal area, and audible moist rales in both lower lungs. After admission, comprehensive laboratory tests were performed, including a complete blood count:white blood cells (WBC) 7.17×10^9^/L, neutrophil percentage (NEU) 88.20%, hemoglobin (Hb) 43 g/L, platelet count (PLT) 307×10^9^/L; reticulocyte percentage (Ret) 11.4%; Coagulation function: D-dimer (D-D) 6.06 mg/L, fibrinogen (FIB) 4.16 g/L; Biochemical blood tests: albumin (ALB) 25.0 g/L, total bilirubin (TBIL) 12.6 μmol/L, alanine aminotransferase (ALT) 12 U/L, aspartate aminotransferase (AST) 12 U/L, alkaline phosphatase (ALP) 104 U/L, gamma-glutamyl transferase (GGT) 77 U/L, lactate dehydrogenase (LDH) 264 U/L, creatine kinase (CK) 49 U/L, creatine kinase isoenzyme (CK-MB) 0.74 ng/ml, blood urea nitrogen (BUN) 12.2 mmol/L, creatinine (CREA) 126 μmol/L (**Table 1**). A comprehensive tumor marker panel in males: neuron-specific enolase (NSE) 17.21 ng/ml. Erythrocyte sedimentation rate (ESR), antinuclear antibody (ANA) profile, antineutrophil cytoplasmic antibody (ANCA) profile, and sputum acid-fast staining showed no abnormalities. Suspected diagnoses included acute lower gastrointestinal bleeding, severe anemia, coagulation dysfunction, alveolar hemorrhage, adrenal hematoma, acute exacerbation of chronic renal insufficiency, hypertension grade 2 (extremely high-risk) and hypoalbuminemia. Treatment measures included multiple blood transfusions, vasoconstriction of visceral blood vessels, acid suppression and gastric protection, gastric lavage with norepinephrine and ice-cold saline, hemostasis with phenyl sulfonamide, oral administration of thrombin powder, anti-infection therapy, nutritional support, and correction of the electrolyte imbalance. However, the patient continued to pass black stools, with positive fecal occult blood test results. Enhanced abdominal computed tomography (CT) revealed a mass in the proximal jejunum with eccentric wall thickening and mild enhancement; multiple low-density nodules and masses in the peritoneal cavity, retroperitoneum, and right adrenal gland with mild heterogeneous enhancement; multiple small nodules in the liver parenchyma with mild enhancement; thickening of the left adrenal gland; left renal atrophy; and multiple cysts in both kidneys (**Figure 1**). Thoracic computed tomography: right upper lobe inflammation, small nodules in the left lower lobe, no evidence of primary pulmonary tumor. The patient presented with recurrent melena and hemoglobin levels fluctuating between 35–53 g/L. On the sixth day of hospitalization, diagnostic paracentesis revealed non-clotted blood, indicating intra-abdominal hemorrhage accompanied by hemodynamic instability and intermittent ventricular tachycardia. After multidisciplinary consultation to evaluate the necessity and risks of surgical exploration, the patient underwent exploratory laparotomy under general anesthesia with family consent. Intraoperative findings revealed a massive intra-abdominal hematoma measuring approximately 1000 mL. A soft mass measuring 4 × 4 cm with surface ulceration and hemorrhage was observed 20 cm from the Cai’s ligament. Three hard masses, each approximately 2–3 cm in size, were palpated in the lumen of the small intestine. Enlarged lymph nodes with hematomas were palpable in the mesentery. A perforated small intestinal tumor with hemorrhage was suspected. The gastrocolic ligament was opened, revealing a massive retroperitoneal hematoma on the right side without exudation. Liver exploration revealed multiple hard nodules in the left hepatic lobe, suggestive of metastatic tumors. To localize the source of the small intestinal bleeding, partial small bowel resection with complex adhesiolysis and duodenojejunal anastomosis was performed (**Figure 2A**).

**Table 1:** Laboratory examination results and reference ranges.

Parameter	Abbreviation	Result	Reference range
White blood cells	WBC	7.17×10^9^/L	4.0–10.0×10^9^/L
Neutrophil percentage	NEU	88.20%	50%–70%
Hemoglobin	Hb	43 g/L	120–160 g/L (male)
Platelet count	PLT	307×10^9^/L	100–300×10^9^/L
Reticulocyte percentage	Ret	11.4%	0.5%–1.5%
D-dimer	D-D	6.06 mg/L	<0.5 mg/L
Fibrinogen	FIB	4.16 g/L	2.0–4.0 g/L
Albumin	ALB	25.0 g/L	35–55 g/L
Total bilirubin	TBIL	12.6 μmol/L	3.4–17.1 μmol/L
Alanine aminotransferase	ALT	12 U/L	5–40 U/L
Aspartate aminotransferase	AST	12 U/L	8–40 U/L
Lactate dehydrogenase	LDH	264 U/L	120–250 U/L
Blood urea nitrogen	BUN	12.2 mmol/L	3.2–7.1 mmol/L
Creatinine	CREA	126 μmol/L	53–106 μmol/L (male)

Postoperatively, the patient was transferred to the intensive care unit for treatment. Subsequent complications included airway bleeding, intermittent hematochezia, and bloody peritoneal drainage, indicating active retroperitoneal hemorrhage. Pathological findings confirmed a malignant tumor of the jejunum, microscopic pathology and immunohistochemical evidence suggested EES. The tumor invaded the mucosal layer of the intestinal wall and extended into the perienteral adipose tissue, with no tumor cells observed at the two ends of the intestinal wall. Eight lymph nodes were identified, four of which showed metastasis (4/8). Pathological microscopic revealed small round cell tumor with round or oval nuclei, scarce cytoplasm, scattered mitosis and necrosis (**Figure 2B**). Immunohistochemistry revealed LCA (-), CK broad-spectrum (-), FLI1 (+)(**Figures 2C, D**), CD99 (+)(**Figures 2E, F**), Ki-67 positive index approximately 80%, P53 (-), Vimentin (+),Dog-1 (-), Desmin (-), CD34 (-), CD117 (-), S100 focal (+), SMA (-), Syn (+); Melan-A focal (+), HMB45 (-),CK7 (-), CK20 (-), CDX-2 (-), CR (-), MC (-), TTF-1 (+), Villin (-), CD56 (-), MSH2 (+), MSH6 (+), MLH1 (+), and PMS2 (+).

On the 9th day of hospitalization, angiography was performed again for hemostatic intervention. Intraoperative exploration revealed normal courses of the femoral artery, abdominal aorta, bronchial artery, superior mesenteric artery, celiac trunk artery, gastroduodenal artery, hepatic artery, and portal vein, with no contrast agent leakage. Jejunal vessels were not visualized, likely due to the surgical intervention. Left renal artery occlusion and right renal artery stenosis at the origin were noted, with no contrast agent leakage or significant bleeding from the cranial vessels. The patient also exhibited elevated levels of inflammatory markers. Bronchoalveolar lavage fluid was collected and sent for pathological examination: A large number of red blood cells, a small number of inflammatory cells and degenerated squamous epithelial cells were observed, with no cancer cells detected. Combined with chest imaging and etiological tests, pulmonary infection was suspected, and imipenem plus tigecycline was administered to treat the infection. The patient remained conscious but showed no improvement in abdominal hemorrhage, with persistently low hemoglobin levels (45 g/L). A follow-up abdominal CT scan was performed on day 12, and consultation with the general surgery department was arranged to consider the formation of a hematoma around the duodenal stump with possible surrounding bleeding. A puncture and drainage procedure is recommended if necessary. On day 19, the patient developed worsening respiratory status, progressive decline in blood pressure and heart rate, and elevated blood lactate levels. Vasoactive drugs were administered to maintain blood pressure, and sodium bicarbonate was used for acid correction. The family declined invasive resuscitation and continued treatment, and the patient eventually died.

## Discussion

EES is a rare, highly malignant, small, round cell tumor that accounts for approximately 10–20% of tumors within the Ewing sarcoma family. It predominantly affects adolescents and young adults, with a relatively low incidence in elderly patients (2), and its clinical manifestations are non-specific. The incidence is slightly higher in men than in women. EES can arise at any anatomical site, but most commonly involves soft tissues, such as the chest wall, extremities, and retroperitoneum. Since Horie et al. first reported a case in 2000, approximately 30 cases have been reported worldwide (3). Yagnik et al. analyzed 31 patients with small intestinal EES and found that the ileum was the most frequently involved segment, whereas jejunal involvement was rare, with only seven cases reported in the literature (4).EES typically exhibits rapid, insidious growth with clinical presentations that are largely dependent on tumor location and size, as no distinct specific features are observed. Common manifestations include local masses, pain, and compressive symptoms, in addition to systemic symptoms such as fever, anorexia, and weight loss (5). In the present case, the patient primarily presented with gastrointestinal bleeding and anemia, which mimicked common gastrointestinal disorders and led to a misdiagnosis. A key characteristic of EES is its propensity for vascular invasion and hematogenous metastasis. The tumor grows rapidly and can invade and disrupt the vascular walls, resulting in rupture and bleeding from either the primary tumor or metastatic lesions. Metastases to sites, such as the liver and retroperitoneum, may also invade blood vessels and contribute to bleeding (6).

In this case, the patient was admitted with prominent multisite hemorrhage, including gastrointestinal bleeding, adrenal hematoma, and hemoptysis. For frail elderly patients, the initial screening should extend beyond a single lesion to include a comprehensive systemic evaluation. In the current case, the initial lactate dehydrogenase and reticulocyte counts were within normal ranges; urinary urobilinogen and bilirubin levels were not significantly elevated; and no abnormalities were detected in the platelet count, antineutrophil cytoplasmic antibodies, antinuclear antibodies, or the coagulation-fibrinolysis system. Hemolysis and hematopoietic disorders were ruled out, as were rheumatic immune diseases such as antiphospholipid antibody syndrome. The multisite hemorrhage was considered a malignant manifestation of extensive tumor invasion into multiple anatomical sites, with subsequent vascular erosion combined with coagulation factor depletion secondary to infection and bleeding. For EES-induced bleeding, management strategies should be tailored to the location and severity of the bleeding, including hemostatic agents, vascular interventional therapy, and surgical hemostasis. However, in patients with advanced cancer and widespread metastases, hemostatic treatment is often ineffective (7).

Regarding differential diagnosis, EES is challenging to identify in its early stages and preoperatively, and is often indistinguishable from benign lesions such as venous malformations, soft tissue abscesses, and adenomas. CT and magnetic resonance imaging (MRI) can delineate the location, size, and morphology of the tumor, as well as its relationship with adjacent tissues, whereas positron emission tomography-CT facilitates the detection of distant metastases (8). Violon et al. described the CT and MRI features of eight EES cases. On CT, tumors appeared as isodense, heterogeneous, non-calcified masses, with or without hemorrhage and necrosis. On MRI, they exhibited isointense T1-weighted signals with heterogeneous enhancement and hyperintense T2-weighted signals with heterogeneous enhancement, except in areas of hemorrhage and necrosis (9).

The definitive diagnosis of EES relies primarily on pathological examination. Characteristic immunohistochemical markers (e.g., strong CD99 expression) and molecular genetic testing for *EWSR1* gene rearrangements serve as gold standards for diagnosis (10). In the present case, surgical specimens were obtained for immunohistochemical analysis (11), which revealed diffuse CD99 (+), LCA (-), FLI1 (+), broad-spectrum CK(-), Ki-67 positive index approximately 80%, P53 negativity, and focal vimentin (+). A rigorous differential diagnosis was performed to rule out other small round cell tumors, including lymphoma (positive for LCA, CD20, CD3, and other lymphocyte markers), neuroendocrine carcinoma (positive for CD56, Syn, CgA, and other neuroendocrine markers), poorly differentiated carcinoma (positive for epithelial markers such as CK), synovial sarcoma (positive for TLE1, EMA, and other markers), and desmoplastic small round cell tumor (positive for vimentin, desmin, and other markers) (12, 13). The diagnosis of EES was confirmed based on the patient’s clinical background, imaging findings, and pathological results. Unfortunately, because of the patient’s critical and complex condition, unstable vital signs, and multiple-organ dysfunction, EWSR1 molecular testing could not be performed to validate the diagnosis, a main limitation of this case. A previous report described a case of primary duodenal EES detected using 18F-FDG PET/CT, which revealed a duodenal mass and multiple hypermetabolic mesenteric lymph nodes with increased radiotracer uptake in tumor-involved regions, facilitating early localization of the primary tumor and its metastases (14).

The management of EES is centered on surgical resection combined with comprehensive therapies, such as chemotherapy and radiotherapy (15). Houdek et al. analyzed 36 patients with EES (mean age, 30 years) who underwent surgical resection alone or surgical resection plus radiotherapy, all of whom received chemotherapy. No significant differences were observed in overall survival, 10-year local recurrence rates, or metastasis-free survival between the two treatment groups (16). Prognostic factors for EES include tumor size, location, stage, and treatment regimen. Chemotherapy is the mainstay of treatment for patients with advanced or metastatic disease, with the most commonly used agents being vincristine, doxorubicin, cyclophosphamide/ifosfamide, and etoposide (17). Radiotherapy is preferentially used for local control in patients with unresectable primary lesions, inadequate surgical margins, or poor response to chemotherapy (18). However, careful risk-benefit assessment is mandatory when considering these therapies in elderly patients. In the present case, the patient was diagnosed with EES accompanied by multiple-organ metastases, indicating an advanced tumor stage. The patient had hemodynamic instability requiring continuous hemostatic therapy, fluid resuscitation, and blood transfusion, with an Eastern Cooperative Oncology Group performance status score of 4. A comprehensive multidisciplinary team evaluation determined that the patient was intolerant of the toxic side effects of chemotherapy, such as myelosuppression and gastrointestinal reactions, which could exacerbate bleeding and be potentially life-threatening. After thorough communication with the patient’s family regarding the benefits and risks of chemotherapy, they declined chemotherapy and opted for conservative care. Therefore, systemic chemotherapy was not administered.

This case highlights that elderly patients often cannot undergo standard oncological treatment because of underlying comorbidities, impaired organ function, and poor treatment tolerance, necessitating individualized assessment and treatment adjustment. In particular, for elderly patients with advanced EES, clinical intervention poses significant challenges, and palliative care may be a more appropriate approach that focuses on symptom relief and quality of life improvement. In recent years, targeted therapy and immunotherapy targeting specific molecular pathways (e.g., the IGF-1R and mTOR pathways) have been actively investigated and may offer promising therapeutic options for future management.

## Conclusion

EES is a rare, highly malignant, small, round cell tumor for which early recognition and prompt pathological diagnosis are critical. This case emphasizes that for elderly patients presenting with unexplained bleeding that is refractory to conventional treatment, the differential diagnosis should be expanded to include rare malignant tumors, such as EES. In the diagnosis and management of this critically ill patient with complex EES, multidisciplinary collaboration played a pivotal role in clarifying the potential causes of multisite hemorrhage, evaluating the preoperative surgical feasibility, obtaining rapid intraoperative pathology, localizing postoperative bleeding, and managing multiple-organ complications. Additionally, close monitoring of vital signs, the presence of shifting dullness on abdominal examination, and non-clotting blood on abdominal paracentesis provided important clinical clues that offered a critical window for surgical exploration. Elderly patients with EES often present with insidious onset, multiple comorbidities, and reduced physiological reserve, leading to delayed diagnosis at an advanced tumor stage with multiple-organ involvement and poor prognosis. In this case, misdiagnoses and missed diagnoses were avoided, and the patient’s survival time was prolonged. In the future, establishing efficient and close multidisciplinary collaboration will be crucial for the comprehensive management, treatment decision-making, and prognostic improvement of such complex and critically ill patients.

## Data Availability

The original contributions presented in the study are included in the article/supplementary material. Further inquiries can be directed to the corresponding author.
